# Predictive value of brachial-ankle artery pulse wave velocity to heart failure with preserved ejection fraction in hospitalised patients with acute dyspnoea

**DOI:** 10.12669/pjms.313.6833

**Published:** 2015

**Authors:** Tao Cong, Shasha Jiang, Ke Wang, Lei Zhong, Jian Wu, Dechun Su

**Affiliations:** 1 Tao Cong, MD. Department of Cardiology, the First Affiliated Hospital of Dalian Medical University, Dalian, Liaoning, 116011 China; 2 Shasha Jiang, MD. Department of Cardiology, The Second Hospital of Dalian Medical University, Dalian, Liaoning, 116027 China; 3 Ke Wang, MD, PhD. Department of Cardiology, the First Affiliated Hospital of Dalian Medical University, Dalian, Liaoning, 116011 China; 4 Lei Zhong, MD. Department of Cardiology, the First Affiliated Hospital of Dalian Medical University, Dalian, Liaoning, 116011 China; 5 Jian Wu, MD. Department of Cardiology, the First Affiliated Hospital of Dalian Medical University, Dalian, Liaoning, 116011 China; 6 Dechun Su, MD, PhD. Department of Cardiology, the First Affiliated Hospital of Dalian Medical University, Dalian, Liaoning, 116011 China

**Keywords:** Heart failure, Differential diagnosis, Pulse wave velocity, Diastolic dysfunction

## Abstract

**Objective:**

To explore the predictive value of the brachial-ankle artery pulse wave velocity (baPWV) for heart failure with preserved ejection fraction (HFpEF).

**Methods:**

Echocardiographic data, B-type natriuretic peptide (BNP) level, and baPWV were assessed in 111 consecutive patients admitted for acute dyspnea. The patients were divided into the HFpEF group (n=71) and the control group (n=40).

**Results:**

Multivariate logistic regression analyses revealed that the ratio of the early mitral inflow velocity to the tissue Doppler velocity (E/e’) at the lateral mitral annulus, BNP, and baPWV were independently predictive of HFpEF. Adding the baPWV to E/e’ at the lateral annulus and to the BNP resulted in an increase in the area under the curve (AUC) to 0.855 (vs. lateral E/e’ alone, P=0.02) or 0.880 (vs. BNP alone, P=0.02), respectively. The AUC of the three combining indicators including the lateral E/e’, BNP, and baPWV was 0.910 (vs. E/e’ lateral alone, P<0.001; vs. BNP alone, P=0.001). The diagnostic accuracy was improved significantly after adding the baPWV to the diagnostic criteria of the 2007 ESC consensus statement (net reclassification improvement 0.127, P=0.02).

**Conclusions:**

Adding the baPWV to the current diagnostic indicators of the 2007 ESC consensus statement could increase the accuracy of predicting HFpEF.

## INTRODUCTION

Despite the better left ventricular ejection fraction (LVEF), heart failure with preserved ejection fraction (HFpEF) has a prognosis similar to or slightly better than heart failure with reduced EF, with a mortality rate of approximately 12-22% in one year.^1^,^2^ How to rapidly diagnose HFpEF in patients hospitalized because of breathing difficulties is practically significant but challenging. No single biomarker or echocardiographic parameter for heart failure is considered adequately sensitive and specific for HFpEF screening. It is expected that multiple markers will need to be combined to yield adequately accurate classification.

There is an association between artery and ventricle stiffness, which play important or even independent roles in the development of cardiac diastolic functions.^3^,^4^ The addition of pulsatile hemodynamic indictors measured using invasive measures to the diagnostic criteria of HFpEF could increase the diagnostic accuracy of the criteria.^5^ Brachial-ankle artery pulse wave velocity (baPWV) is a simple, inexpensive, non-invasive, and reproducible arterial stiffness measurement method. It is used extensively in basic medical institutions in China. However, the values of this indicator in HFpEF are still unclear.

This study was performed to evaluate the diagnostic properties of baPWV against and in combination with commonly used echocardiographic parameters in hospitalized patients with acute dyspnea.

## METHODS

A total of 239 consecutive patients from the heart failure department at our hospital (Dalian, China) presenting between April 2012 and September 2013 with the symptoms of acute dyspnea were included in this prospective study. Acute HFpEF was defined by the combination of acute dyspnea, clinical and radiographic signs of pulmonary edema, favorable responses to intravenous loop diuretics and nitrates within 24 hours, B-type natriuretic peptide (BNP) ≥200 pg/mL, and a LVEF ≥50% on admission.^6^ For all participants, a coronary angiogram or coronary CT angiogram was performed to diagnose the patients with coronary artery disease. We excluded the patients with recent acute coronary syndrome, valve disease more extensive than mild, LV systolic dysfunction, congenital heart disease, primary or secondary myocardial disease, restrictive pericardial disease, renal failure, and high-output disease. The patients with neither heart failure nor the above diseases were the control group.

### Assessment of brachial-ankle artery pulse wave velocity

Arterial stiffness was evaluated in terms of baPWV by a single observer using an automatic waveform analyzer (model VaSera VS-1000; Fukuda Denshi Co. Ltd, Peking, China) within 6 hours after admission. The patient rested in the supine position at room temperature (22-25°C). BP cuffs were applied above the medial malleolus of both legs and above the elbows of both arms. The device used the height of the subject to calculate path length from the aortic arch to the brachial sensor (d1) and that from the aortic arch to the ankle sensor (d2). The time interval (t) between the initial rise of the pulse pressure waveforms at the brachial and the tibial arteries was measured, and baPWV was calculated using the following equation: baPWV (cm/s) = (d2−d1)/t.^7^

### Echocardiography

A detailed 2-dimensional and Doppler echocardiogram was performed according to the recommendations of the guideline.^8^ Echocardiographs were performed by two independent investigators using a Vivid 7 digital color Doppler ultrasound (GE Vingmed Ultrasound AS, Horten, Norway) at the time of therapy initiation, immediately before or after measurement of baPWV. The tissue Doppler imaging (TDI) of the mitral annulus movement was obtained from the apical four-chamber view. A 1.5-mm sample volume was placed sequentially at the septal and lateral border of the mitral annulus, where we obtained the systolic (s’) and the early (e’) and late (a’) diastolic peak velocities.

### Natriuretic peptides

BNP was measured using a PBNP Flex Kit (Dade Behring Inc., Shanghai, China) according to the manufacturer’s instructions.

### Statistical analysis

Descriptive statistics were given as mean, standard deviation, frequency and percentage. The unpaired t-test was used to assess the differences in continuous variables. The chi-squared test was used to assess the differences in percentage data. Receiver operating characteristic (ROC) curves were constructed to illustrate the diagnostic performance of measures of baPWV and of echocardiographic parameters. The area under the ROC curve (AUC) and z-statistic were used to compare all methods. The multiple stepwise logistic regression was applied to determine the most effective combination of diagnostic tests for predicting the HFpEF. Net reclassification improvement (NRI), compared with echocardiographic parameters recommended by a consensus statement on the diagnosis of HFpEF by the ESC,^9^ was calculated according to the method of Pencina et al.^10^ A *P*-value of <0.05 was considered statistically significant. All data were analyzed by SPSS 20.0 and MedCalc 12.

## RESULTS

### Study population and baseline characteristics

The study enrolled 239 consecutive patients with acute dyspnea. Seventy-one patients were diagnosed with HFpEF, 128 were excluded, and the remaining 40 patients without heart failure were included in the control group.

Both groups were similar with respect to age or gender. Compared to the control group, the BMI in the HFpEF group was slightly higher; the percentage of combined hypertension, diabetes mellitus, and atrial fibrillation was higher; and the use of calcium channel blockers, ACEIs, and/or ARBs was also much higher (Table-I).

**Table-I:** Baseline characteristics of patients.

Demographics	Controls (n=40)	HFpEF (n=71)	P-value
Age (years)	63±15	65±9	0.469
Female (%)	17 (42.5)	36 (50.7)	0.406
Body mass index (kg/m^2^)	25.4±2.4	27.0±2.5	0.002
Coronary artery disease (%)	9 (22.5)	25 (35.2)	0.163
Hypertension (%)	25 (62.5)	59 (83.1)	0.015
Diabetes mellitus (%)	8 (20.0)	38 (53.5)	0.001
Atrial fibrillation (%)	5 (12.5)	22 (31.0)	0.029
Peripheral arterial disease (%)	2 (5.0)	6 (8.5)	0.709
Chronic obstructive pulmonary disease (%)	6 (15.0)	11 (15.5)	0.945
NYHA class II		21 (29.6)	
NYHA class III		28 (39.4)	
NYHA class IV		22 (31.0)	
ACEI and/or ARB (%)	16 (40)	54 (76.1)	<0.001
Beta-blocker (%)	20 (50)	44 (62.0)	0.220
Digoxin (%)	1 (2.5)	8 (11.3)	0.153
Calcium channel blocker (%)	23 (57.5)	55 (77.5)	0.023
Spironolactone (%)	1 (2.5)	11 (15.5)	0.053
SBP (mmHg)	145.7±23.1	159.6±23.3	0.003
DBP (mmHg)	78.8±15.7	86.7±15.5	0.011

All values are the means ± SD or n (%). HFpEF, heart failure with preserved ejection fraction; ACEI, angiotensin-converting enzyme inhibitor; ARB, angiotensin receptor blocker; SBP, systolic blood pressure; DBP, diastolic blood pressure.

### Diagnostic indicators of HFpEF

Patients with HFpEF had greater wall thickness, LV mass index (LVMI), and left atrial volume index than the control group. The E and Ard-Ad of the traditional Doppler indicators in the HFpEF group were also higher than those in the control group (all P < 0.05). Regardless of sampling location (septal, lateral wall, or mean), E/e’ significantly increased in the HFpEF group (all *P* < 0.01). The BNP and baPWV in the HFpEF group were also higher than those in the control group (both P<0.0001). The differences in other 2-dimensional, Doppler, and tissue Doppler indicators between these two groups all did not reach statistical significance (all P > 0.05) (Table-II).

**Table-II:** Diagnostic parameters of the two group patients.

	Controls (n=40)	HFpEF (n=71)	P-value
LAVI (mL/m^2^)	32.9±10.1	39.9±9.3	0.001
LVEDVI (mL/m^2^)	44.6±9.8	46.1±7.1	0.398
IVST (mm)	10.6±1.6	12.1±1.6	<0.001
PWT (mm)	10.0±1.8	10.7±1.3	0.032
LVMI (g/m^2^)	99.4±13.9	110.3±19.0	0.001
LVEF (%)	67.5±6.2	67.3±6.2	0.884
E (cm/s)	76.0±19.4	83.8±39.2	0.163
A (cm/s)	80.7±15.2	95.0±40.4	0.057
E/A	0.96±0.43	0.94±0.57	0.907
DT (ms)	212.7±62.5	227.9±60.1	0.210
Ard-Ad (ms)	13.7±9.0	28.4±23.9	0.003
E/e’ septal	11.5±3.6	14.8±5.8	0.001
E/e’ lateral	8.6±3.2	12.8±4.9	<0.001
E/e’ average	9.9±3.2	13.8±5.1	<0.001
BNP (pg/mL)	144.2±92.4	283.2±164.0	<0.001
baPWV (m/s)	9.4±2.5	12.8±3.4	<0.0001

All values are the means ± SD. LAVI, left atrial volume index; IVST, interventricular septal thickness; PWT, posterior wall thickness; LVEDVI, left ventricular end-diastolic volume index; LVMI, left ventricular mass index; LVEF, left ventricular ejection fraction; E, early mitral flow velocity; A, late mitral flow velocity; DT, deceleration time; Ard-Ad, difference between duration of reversed pulmonary vein atrial systole flow and duration of mitral A wave flow; e’, early mitral annulus velocity; E/e’, the ratio of mitral inflow velocity and early mitral annulus velocity. BNP, B-type natriuretic peptide; baPWV, brachial-ankle artery pulse wave velocity.

### Diagnostic performance and ROC analysis

**Fig.1:**
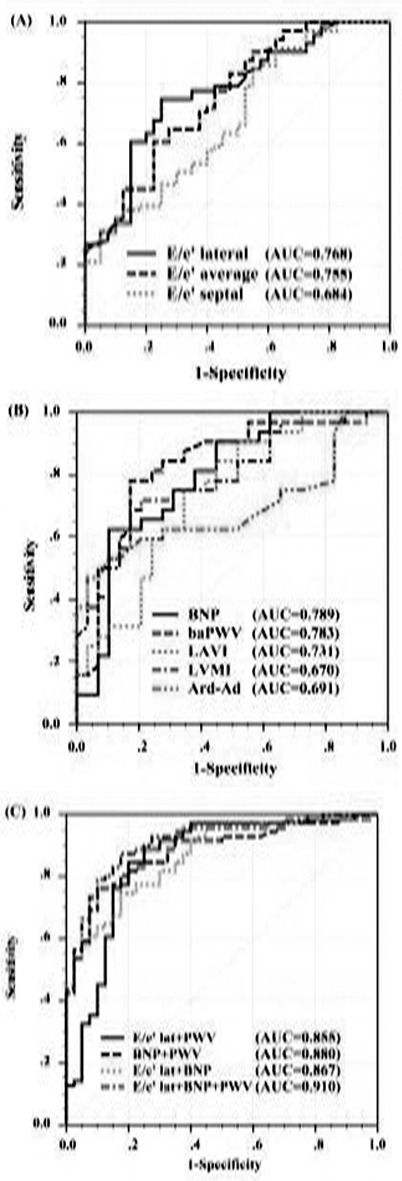
Receiver operating characteristic curves for single parameter and combinations of three parameters in differentiating between HFpEF and no HFpEF.

The diagnostic value of the different parameters for HFpEF diagnosis are shown in Table-III. Fig.1 shows that the AUCs of the lateral and average E/e were higher than the septal E/e (P < 0.0001 and P = 0.01, respectively). Although a lateral E/e’>15 and septal E/e’>15 were recommended by the 2007 ESC consensus as a main diagnostic parameter, they had high specificity (92.5% and 87.5%, respectively) but low sensitivity (32.6% and 39.1%, respectively). LAVI, at ≥34 mL/m^2^, showed the best diagnostic accuracy among the remaining echocardiographic parameters. The AUCs of serum BNP and baPWV were essentially equivalent to that of lateral E/e’ or even better (Fig.1). A cut-off value of ≥ 11.38 m/s for baPWV was obtained from the ROC curve for predicting the HFpEF patients (Table-III).

**Table-III:** Diagnostic accuracy in identifying heart failure patients with preserved ejection fraction.

	Sensitivity (%)	Specificity (%)	PPV (%)	NPV (%)
LAVI≥34 mL/m^2^	66.2	72.5	81	54.7
LVMI>149 g/m^2^ (male) or >122 g/m^2^ (female)	19.7	97.5	93.3	40.6
DT>280 ms and E/A<0.5	13.3	100	100	57.4
Ard-Ad >30 ms	34.4	96.6	91.7	57.1
E/e’≥12 (lateral)	60.6	87.5	89.6	55.6
E/e’≥13 (average)	56.3	82.5	85.1	51.6
E/e’>15 (septal)	42.3	77.5	76.9	43.1
E/e’>15 (lateral)	35.2	92.5	89.3	44.6
E/e’>15 (average)	40.8	87.5	85.3	45.5
BNP≥200 pg/mL	69	70	80.3	56
baPWV≥11.38 m/s	69	80	86	59.3

PPV, positive predictive value; NPV, negative predictive value.

### Multivariable models

Using the clinical diagnosis of heart failure as a dependent variable, lateral E/e’, BNP, LAVI, LVMI, Ard-Ad, and baPWV were used to perform the multivariate stepwise logistic regression analysis. Finally, the variables left in the equation were lateral E/e’ (OR=1.27, 95% CI 1.04-1.56, P=0.02), BNP (OR=1.77, 95%CI 1.05-3.0, P=0.03), and baPWV (OR=2.26, 95% CI 1.15-4.44, P=0.02). ROC curves were plotted using the combination of baPWV and lateral E/e’, the combination of baPWV and BNP, and the combination of these three. Compared with those of the simple lateral or simple BNP, the AUCs of the combination of indicators were larger (all P<0.05). The increasing trend of the combination of these three indicators was the most significant (P<0.01) (Fig.1).

### Incremental diagnostic value—NRI

When we used all echocardiographic parameters with cutoff values as suggested in the 2007 ESC consensus,^9^ 88 patients were classified correctly and 23 incorrectly. Adding baPWV led to a highly significant NRI of up to 11.6%. The correct classifications increased to 97 people, and the incorrect classifications decreased to 14 people.

## DISCUSSION

The major indicators of the 2007 ESC consensus for diagnosis of HFpEF include lateral or mean E/e’, natriuretic peptide, LAVI, LVMI, Ard-Ad, and atrial fibrillation. The E/e’ at lateral annulus had the best diagnostic effect compared with other locations. Multivariate logistic regression results confirmed that lateral E/e’, BNP, and baPWV had independent predictive value for heart failure. The AUCs of baPWV and the aforementioned combination of indicators were significantly better than that of the single indicator, indicating that baPWV increased the diagnostic effects of lateral E/e’ and BNP. We further used baPWV as an auxiliary indicator and directly introduced it into the 2007 ESC consensus; the results showed that although baPWV caused one new misdiagnosis of heart failure, it also recognized seven cases of heart failure that were previously missed. In exchange for a slightly decreased specificity, the sensitivity significantly improved and the overall diagnostic effect significantly increased.

We did not exclude patients with coronary artery disease from our data because HFpEF and CAD frequently coexist.^11^ Patients with acute unstable coronary syndromes were excluded because the diagnosis and treatment of these patients are clearly different from those of general HFpEF. Although the percentages of patients with and without CAD were not substantially different between the HFpEF and control groups, we could not exclude the influences of stable coronary heart disease on the experimental results. However, when these patients were excluded from the analysis, the diagnostic performance of measures of pulsatile function improved possibly.^12^

Hypertension is one of the major reasons for increased arterial stiffness and leads to diastolic dysfunction. However, CAD, diabetes mellitus, obesity,^13^ and even hypertrophic cardiomyopathy are also associated with arterial stiffness.^14^ Patients with more factors of metabolic syndromes have faster PWV,^15^ indicating that risk factors that constitute metabolic syndrome could have cumulative effects on arterial stiffness. Although 62.5-83.1% of patients had hypertension in this study, the HFpEF group had a higher BMI and higher rate of diabetes mellitus. Therefore, the higher values of baPWV in the HFpEF group does not depend entirely on hypertension.

In China, the most-used PWV methods include carotid-femoral PWV and baPWV; the latter is more popular. The baPWV incorporates both central and peripheral segments of the arterial tree. In a study of 409 healthy adults, aortic PWV was the primary independent correlate of baPWV, explaining 58% of the total variance in baPWV. An additional 23% of the variance was explained by leg PWV.^12^ Currently, it is debated whether central or baPWV is better for evaluation of ventricular diastolic function. One study indicated that central but not lower-extremity PWV correlated with patients’ transition from acute decompensated heart failure to chronic compensated heart failure.^16^ Another study demonstrated that baPWV correlated better with LV mass and diastolic function than carotid-femoral PWV.^17^ The central arterial stiffness index measured using invasive measures has complementary diagnostic value for HFpEF.^5^ The conditions of the patients enrolled in the current study were more severe. We found that the non-invasive indicator baPWV, which combined central and peripheral arterial stiffness, had more differential significance for patients who were hospitalized due to acute dyspnea. The ease of use and promotion value of baPWV cannot be competed with interventional technology and central artery PWV.

Non-invasive measurements of large artery stiffness index mainly include pulse pressure/pulse pressure index, PWV, augmentation index, dilation coefficient, or compliance coefficient.

### Limitations of the study

The main limitation of this study was that the HFpEF-diagnostic values of the above indicators were not comprehensively compared. Some of the above indicators require ultrasonography or MRI measurement, and they require skilled, experienced operators and have a high cost. They are not suitable for promotion in China. The limitations of the present study also include its relatively small population size and the use of a comprehensive clinical diagnosis as the diagnostic criterion for HFpEF, without an invasive marker to confirm LV end-diastolic pressure.

## CONCLUSIONS

The baPWV is a promising, non-invasive procedure to complement E/e’ at the lateral mitral annulus, BNP, and the 2007 ESC consensus statement for the diagnosis of HFpEF in hospitalized patients with acute dyspnea. However, more studies are needed to become a regular practice to compliment existing diagnostic tools as given in 2007 ESC consensus statement.

## References

[1] Bhatia RS, Tu JV, Lee DS, Austin PC, Fang J, Haouzi A (2006). Outcome of heart failure with preserved ejection fraction in a population-based study. N Engl J Med.

[2] (2012). Meta-analysis Global Group in Chronic Heart F. The survival of patients with heart failure with preserved or reduced left ventricular ejection fraction: an individual patient data meta-analysis. Eur Heart J.

[3] Tartiere-Kesri L, Tartiere JM, Logeart D, Beauvais F, Cohen Solal A (2012). Increased proximal arterial stiffness and cardiac response with moderate exercise in patients with heart failure and preserved ejection fraction. J Am Coll Cardiol.

[4] Russo C, Jin Z, Palmieri V, Homma S, Rundek T, Elkind MS (2012). Arterial stiffness and wave reflection: sex differences and relationship with left ventricular diastolic function. Hypertension.

[5] Weber T, Wassertheurer S, O’Rourke MF, Haiden A, Zweiker R, Rammer M (2013). Pulsatile hemodynamics in patients with exertional dyspnea: potentially of value in the diagnostic evaluation of suspected heart failure with preserved ejection fraction. J Am Coll Cardiol.

[6] Arques S, Roux E, Sbragia P, Ambrosi P, Pieri B, Gelisse R (2005). B-type natriuretic peptide and tissue Doppler study findings in elderly patients hospitalized for acute diastolic heart failure. Am J Cardiol.

[7] Amoh-Tonto CA, Malik AR, Kondragunta V, Ali Z, Kullo IJ (2009). Brachial-ankle pulse wave velocity is associated with walking distance in patients referred for peripheral arterial disease evaluation. Atherosclerosis.

[8] Lang RM, Bierig M, Devereux RB, Flachskampf FA, Foster E, Pellikka PA (2005). Recommendations for chamber quantification: a report from the American Society of Echocardiography's Guidelines and Standards Committee and the Chamber Quantification Writing Group, developed in conjunction with the European Association of Echocardiography, a branch of the European Society of Cardiology. J Am Soc Echocardiogr.

[9] Paulus WJ, Tschope C, Sanderson JE, Rusconi C, Flachskampf FA, Rademakers FE (2007). How to diagnose diastolic heart failure: a consensus statement on the diagnosis of heart failure with normal left ventricular ejection fraction by the Heart Failure and Echocardiography Associations of the European Society of Cardiology. Eur Heart J.

[10] Pencina MJ, D’Agostino RB, D’Agostino RB, Vasan RS (2008). Evaluating the added predictive ability of a new marker: from area under the ROC curve to reclassification and beyond. Stat Med.

[11] Holland DJ, Prasad SB, Marwick TH (2010). Contribution of exercise echocardiography to the diagnosis of heart failure with preserved ejection fraction (HFpEF). Heart.

[12] Sugawara J, Hayashi K, Yokoi T, Cortez-Cooper MY, DeVan AE, Anton MA (2005). Brachial-ankle pulse wave velocity: an index of central arterial stiffness?. J Hum Hypertens.

[13] Goto T, Ohte N, Fukuta H, Wakami K, Tani T, Kimura G (2013). Relationship between effective arterial elastance, total vascular resistance, and augmentation index at the ascending aorta and left ventricular diastolic function in older women. Circ J.

[14] Boonyasirinant T, Rajiah P, Setser RM, Lieber ML, Lever HM, Desai MY (2009). Aortic stiffness is increased in hypertrophic cardiomyopathy with myocardial fibrosis: novel insights in vascular function from magnetic resonance imaging. J Am Coll Cardiol.

[15] Safar ME, Thomas F, Blacher J, Nzietchueng R, Bureau JM, Pannier B (2006). Metabolic syndrome and age-related progression of aortic stiffness. J Am Coll Cardiol.

[16] Kim DB, Baek SH, Jang SW, Her SH, Shin DI, Park CS (2013). Improvement of arterial stiffness in the transition from acute decompensated heart failure to chronic compensated heart failure. Clin Cardiol.

[17] Yu WC, Chuang SY, Lin YP, Chen CH (2008). Brachial-ankle vs carotid-femoral pulse wave velocity as a determinant of cardiovascular structure and function. J Hum Hypertens.

